# Acute retinal pigment epitheliitis, a diagnostic myth?

**DOI:** 10.1038/s41433-023-02683-w

**Published:** 2023-08-02

**Authors:** Mays Al-Nofal, Peter Charbel Issa

**Affiliations:** 1 Oxford Eye Hospital, Oxford University Hospitals NHS Foundation Trust, Oxford, UK; 2 Nuffield Laboratory of Ophthalmology, Nuffield Department of Clinical Neurosciences, University of Oxford, Oxford, UK

**Keywords:** Retinal diseases, Eye manifestations

Acute retinal pigment epitheliitis (ARPE), also known as Krill’s disease, was first described by Krill and Deutman in 1972 [1] as a self-limiting unilateral or bilateral inflammation of the retinal pigment epithelium. Three of the reported six patients were seen in the acute stage. They described discrete, mostly paracentral clusters of 2–4 round dark greyish spots in the macular region, surrounded by a yellowish-white circular halo-like zone. The lesions faded in some, but persisted in other patients who developed permanent pigmentary changes. The authors hypothesised that lesions observed in the acute phase may represent focal swelling of the pigment epithelium (RPE).

At that time, their impressively detailed report was based on patients’ history, visual acuity testing, and morphological assessment limited to fundoscopy and fluoresceine angiography. In the more recent literature that referred to this initial report, authors had the advantage of using optical coherence tomography (OCT) imaging, sometimes complemented by fundus autofluorescence (FAF) imaging and/or indocyanine green (ICG) angiography, allowing a more detailed phenotypic analysis.

We noticed in discussions with colleagues that some of us never diagnose ARPE and that there seems to be no consensus regarding the exact nature of this condition. This prompted us to review the more recent literature that also includes at least OCT imaging, which consists mostly of case reports and case series. We noticed inconsistencies of the phenotypes reported as ARPE and interpret that these may broadly be allocated to three different groups. While some cases with limited reported clinical data may be within the spectrum of these three groups, few others remain difficult to categorise.

Group 1: Phenotypic features may also be compatible with the diagnosis of multiple evanescent white dot syndrome (MEWDS) [2–7]. These cases show multiple whitish macular lesions and hyperfluorescent dots at the posterior pole on fluorescein angiography. OCT imaging showed foveal ellipsoid zone (EZ) loss that normalised over time. However, most cases are reported without results of additional imaging modalities such as FAF or indocyanine green (ICG) angiography that usually show characteristic features in MEWDS.

Group 2: These cases showed macular changes that may be interpreted within the spectrum of central serous chorioretinopathy (CSC)/pachychoroid disease [1, 7–11]. This mainly includes focal hypo- and hyper-pigmentations which may be observed following CSC.

Group 3: The characteristic of this group is a well-defined hyperreflectivity in the foveal centre with associated loss of reflectivity of the ellipsoid zone [8, 12–17]. These changes are similar to findings found on OCT images in the acute stage after light damage or in eyes with acute macular neuroretinopathy (AMN), although the latter is usually not observed in the central fovea.

With hindsight, the cases described by Krill and Deutman in their initial report on ARPE may also be classified as having CSC/pachychoroid disease, some possibly complicated by secondary MEWDS. Reliable distinction of these entities would not have been possible using retinal imaging methodology available at the time. On the other hand, many cases described as ARPE in the more recent literature often lack or do not mention features described in the original article, including the greyish appearance in the acute stage with surrounding halo, the pigmentary changes, or the paracentral cluster of lesions. Moreover, many cases show monofocal changes limited to the outer aspect of the central foveal photoreceptor layer.

In order to reliably distinguish entities such as CSC, MEWDS, AMN, retinal light damage or acute idiopathic maculopathy, the diagnosis should always be based on multimodal imaging and the full history of the disease course. Inclusion of such detailed information should be a requirement for future reports. This may allow us to learn if specific entities still need to be characterised in full detail. For instance, cases with hyperreflectivity of the foveal outer nuclear layer without a history of light damage might comprise a separate phenotypic entity that warrants further investigation.

In summary, it is plausible that the cases described in the initial report by Krill and Deutman [1] are within the spectrum of other diseases, and the same applies to many cases reported later. ARPE may be a diagnostic relic of the pre-OCT era as there is not sufficient evidence that it is a diagnosis in its own right.

## References

[1] Krill AE, Deutman AF (1972). Acute retinal pigment epitheliitus. Am J Ophthalmol.

[2] Moroz I, Alhalel A (2008). Resolution of retinal pigment epitheliitis—optical coherence tomography findings. Retin Cases Brief Rep.

[3] Merkoudis N, Granstam E (2013). Acute retinal pigment epitheliitis: optical coherence tomography findings at onset and follow-up. Acta Ophthalmol.

[4] Iu LPL, Lee R, Fan MCY, Lam W-C, Chang RT, Wong IYH (2017). Serial spectral-domain optical coherence tomography findings in acute retinal pigment epitheliitis and the correlation to visual acuity. Ophthalmology.

[5] Roy R, Saurabh K, Thomas NR (2021). Multicolor imaging in a case of acute retinal pigment epitheliitis. Retin Cases Brief Rep.

[6] Aydoğan T, Güney E, Akçay BİS, Bozkurt TK, Ünlü C, Ergin A (2015). Acute retinal pigment epitheliitis: spectral domain optical coherence tomography, fluorescein angiography, and autofluorescence findings. Case Rep Med.

[7] Baillif S, Wolff B, Paoli V, Gastaud P, Mauget-Faÿsse M (2011). Retinal fluorescein and indocyanine green angiography and spectral-domain optical coherence tomography findings in acute retinal pigment epitheliitis. Retina.

[8] Cho HJ, Lee DW, Kim CG, Kim JW (2011). Spectral domain optical coherence tomography findings in acute retinal pigment epitheliitis. Can J Ophthalmol.

[9] Kılıç R (2021). Acute retinal pigment epitheliitis: a case presentation and literature review. Arq Bras Oftalmol.

[10] Kim JW, Jang SY, Park TK, Ohn Y-H (2011). Short-term clinical observation of acute retinal pigment epitheliitis using spectral-domain optical coherence tomography. Korean J Ophthalmol.

[11] Hsu J, Fineman MS, Kaiser RS (2007). Optical coherence tomography findings in acute retinal pigment epitheliitis. Am J Ophthalmol.

[12] Hall EF, Ahmad B, Schachat AP (2012). Spectral-domain optical coherence tomography findings in acute retinal pigment epitheliitis. Retin Cases Brief Rep.

[13] De Bats F, Wolff B, Mauget-Faÿsse M, Scemama C, Kodjikian L (2013). B-scan and “en-face” spectral-domain optical coherence tomography imaging for the diagnosis and followup of acute retinal pigment epitheliitis. Case Rep Med.

[14] González Escobar AB, Ibáñez García A, Chinchurreta Capote A, Gismero Moreno S, Lorenzo Soto M (2022). Acute retinal pigment epitheliitis (ARPE). A case report. Arch Soc Esp Oftalmol.

[15] Wu W-C, Li Y-H, Chang Y-C (2019). Spectral-domain optical coherence tomography finding of acute retinal pigment epitheliitis. Taiwan J Ophthalmol.

[16] Wykrota AA, Löw U, Fries FN, Seitz B, Abdin AD (2022). Unilaterale makuläre Pigmentepithelunregelmäßigkeiten bei einem 38-jährigen Patienten—“Akute retinale Pigmentepitheliitis”. Klin Monbl Augenheilkd.

[17] Cho HJ, Han SY, Cho SW, Lee DW, Lee TG, Kim CG (2014). Acute retinal pigment epitheliitis: spectral-domain optical coherence tomography findings in 18 cases. Invest Ophthalmol Vis Sci.

